# *Spiradiclis longanensis*, a new species of Rubiaceae from China

**DOI:** 10.3897/phytokeys.55.4975

**Published:** 2015-08-05

**Authors:** Haizhen Wen, Ruijiang Wang, Shujun Deng

**Affiliations:** 1 Key Laboratory of Plant Resources Conservation and Sustainable Utilization, Guangdong Provincial Key Laboratory of Applied Botany, South China Botanical Garden, the Chinese Academy of Sciences, Guangzhou 510650, Guangdong, China; 2 Medical College, Foshan University, Foshan 528000, Guangdong, China

**Keywords:** China, *Spiradiclis*, Rubiaceae, taxonomy

## Abstract

A new species of *Spiradiclis* (Rubiaceae) was found during our field trip to Guangxi, China, and is described here as *Spiradiclis longanensis* R. J. Wang. This species is readily distinguishable from other prostrate and decumbent species of the genus described previously by dense pubescence all over the plant, 5–20 small flowers per cymose, linear calyx lobes, and tubular corolla. The conservation status of VU was preliminarily assessed according to IUCN categories and criteria.

## Introduction

The genus *Spiradiclis* Blume belongs to the tribe Ophiorrhizeae in the subfamily Rubioideae (Bremekamp 1952, Bremer and Manen 2000), and comprises approximately 45 species (Wang et al. 2015). Its members are perennial herbs or subshrubs and usually grow at the entrances of caves or mountain cliffs in the limestone area of Southern China and Northern Vietnam (Lo 1999, Wang 2002, Chen and Taylor 2011). They favor moist and shaded habitats, usually with ferns, Gesneriaceae, Begoniaceae, and Loganiaceae plants. *Spiradiclis* species are characterized by having usually 5-merous flowers and globose or ovoid capsules in a cymose or paniculiform inflorescence with dichasial or scorpioid axes (Deng et al. 2014).

During a field investigation in 2013, we found a semi-erect and hairy herb, which was similar to *Spiradiclis danxiashanensis* R. J. Wang in habit and leaf size. Unfortunately, the voucher (*R. J. Wang & S. J. Deng 2324*, IBSC) was only a vegetative individual at that time. The specimens with flowers and fruits were eventually collected after our subsequent collecting in different seasons. The 5-merous distylous flowers and ovoid to subglobose capsules in a cymose demonstrated that the plant was a true member of *Spiradiclis*. Our comprehensive examination showed that the flowered and fruited specimens represent a new and undescribed species of *Spiradiclis*.

## Material and methods

All materials were collected by ourselves and deposited at the herbarium of South China Botanical Garden, the Chinese Academy of Sciences (IBSC). The leaf materials were carefully taken from the specimen *R.J. Wang & Q. Liao 2592* (IBSC) and washed three times with 95% ethanol and then mounted on copper stubs. Samples were sputter-coated with gold particles for 15 min using a JEOL JFC-1600 AUTO FINE COATER. Scanning Electron Microscope (SEM) observation was carried out by JEOL JSM-T300. Digital images of the coated leaf surface were taken during the observation.

## Taxonomy

### Spiradiclis longanensis

Taxon classificationPlantaeGentianalesRubiaceae

R. J. Wang sp. nov.

urn:lsid:ipni.org:names:77148932-1

Fig. 1

#### Diagnosis.

*Spiradiclis longanensis* is similar to *Spiradiclis danxiashanensis*, from which it differs by its dense hairs in stems, leaves and inflorescences, (5–)7–10 secondary veins each side, a terminal or upper axillary cymose with 5–20 small flowers, linear calyx lobes, and tubular corolla.

#### Type.

**CHINA.** Guangxi Zhuangzu Autonomous Region, Nanning City, Longan County, Pingshan Town, Tuanjie Village, 22°57’’N, 107°34’E, 219 m, 4 Jun 2014, long-styled flowers, *R. J. Wang 2682* (holotype: IBSC; isotypes: IBSC[6]).

#### Description.

Perennial herbs, prostrate when young and decumbent while growing, rooting at nodes adhering to ground; stems terete, densely pubescent. Stipules linear, 3.8–6.5 mm long. Petioles 0.5–2 cm long, sparsely pubescent; leaf blades oval, ovate to broadly ovate, 1.1–5 (–6.2) × 0.5–3 cm, base cuneate to rounded, apex acute, papery, hairy both sides; secondary veins (5–)7–10 on each side, conspicuous, depressed adaxially and projected abaxially; leaf epidermal cells irregularly polygonal both sides, with sinuous anticlinal walls; stomata paracytic. Inflorescences terminal or upper axillary, cymose, (5–) 10–20-flowered, usually condensed into capitate-like; peduncles pubescent, 5–12 mm long, to 3 cm while fruiting; bracts linear, 6–10 mm long. Flowers distylous, 5-merous; pedicels sparsely pubescent, 1–3 mm long, usually condensed. Hypanthium obconical, 1–2 mm long, sparsely pubescent; lobes 5, linear, 2.5–4 mm long, pubescent. Corollas tubular, white, sparsely hairy abaxially, tubes 2–3 mm long, ca. 1 mm wide; lobes subovate, ca. 1.5 × 1 mm; stamens 5; anthers oblong-linear, ca. 1 mm long; stigmas bilobed; ovary 2-celled, ovules on the axile placentas. Long-styled flowers: a ring of pubescence in throat; stamens below the pubescent ring and included, filaments adnate to the base of corolla tube, ca. 1 mm long; styles exserted, ca. 3 mm long, stigma lobes capitate. Short-styled flowers: corolla tubes densely pubescent inside; stamens extend to the corolla throat but included, filaments adnate to the upper portion corolla tube, ca. 1 mm long; styles included, ca. 1 mm long, stigma lobes ovate. Fruits capsular, ovoid to subglobose, 3–4 mm, hairy, dehiscing loculicidally and then septicidally; valves 4, crustaceous, ca. 3.5 mm long; calyx lobes persistent, 1.8–2 mm long. Seeds ca. 30 per capsule, ca. 0.5 mm long, rectangular pyramid, brown, testa papillate.

**Figure 1.**
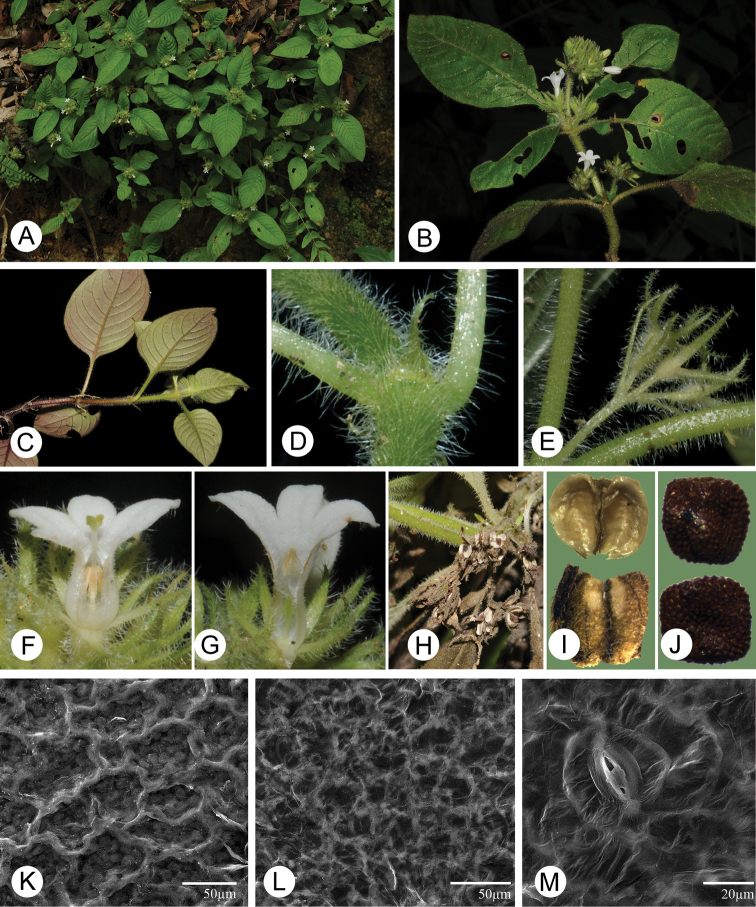
Morphological characters of *Spiradiclis longanensis*. **A** Habit **B** Flowering branch, showing the position of inflorescence **C** Vegetative branch, showing the linear stipules, hairy leaves and venation pattern **D** Hairy stems, petioles, and stipules **E** Hairy young infructescence, showing the linear bracts and calyx lobes **F, G** Longitudinally dissected long- and short-styled flower, respectively, showing the positions of anthers and styles **H** infructescence, showing the dehiscent mature capsules **I** Capsule valves in adaxial (up) and abaxial sides (low), showing the dehiscence pattern **J** Seeds in adaxial (up) and abaxial sides (low) under stereoscope, showing the hilum and papillate surface **K, L, M** Morphology of adaxial, abaxial leaf epidermis, and stomatal apparatus under SEM, respectively (from *R.J. Wang & Q. Liao 2592*). Photos by Ruijiang Wang.

#### Distribution.

Known only from the type locality. The plants grow in the entrances of limestone caves or moist cliffs of hill sides.

#### Ecology.

Flowering in May–June and fruiting in June–August.

#### Conservation status.

The number of *Spiradiclis longanensis* was less than 500 individuals within an area of less than 5 km^2^; however we know that the present field investigation is in a very low density and the evaluation we made here was based on all the available information up to now. We therefore assign a preliminary IUCN threat status of Vulnerable (VU, B2ab(ii, iii, iv); D) to *Spiradiclis longanensis* (IUCN 2001). The populations are probably prone to the effects of human activities or stochastic events in future, because the plants grow nearby the main traffic road or rice field.

#### Additional specimens examined

**(paratypes). CHINA. Guangxi Zhuangzu Autonomous Region**. Longan County, Pingshan Town, Tuanjie village, 5 Apr 2013, *R. J. Wang & S. J. Deng 2324* (IBSC); 6 Oct 2013, *R. J. Wang & Q. Liao 2592* (IBSC), *2595* (IBSC); 18 Feb 2014, *R. J. Wang, S. J. Deng & Q. Liao 2623* (IBSC); 4 Jun 2014, short-styled flowers, *Ruijiang Wang 2683* (IBSC), long-styled flowers, *Ruijiang Wang 2684* (IBSC).

#### Discussion.

Similar to *Spiradiclis longanensis* in this genus, *Spiradiclis danxiashanensis*, *Spiradiclis guangdongensis*, *Spiradiclis hainanensis* and *Spiradiclis umbelliformis* are all in prostrate or decumbent habit, but their leaf laminas, inflorescences, calyx lobes, and flowers are different from *Spiradiclis longanensis* and can easily be distinguished. The latter four species usually have rounded, orbicular, or ovate leaf laminas in 1–2.5 mm long and with 3–5 unobvious secondary veins each side, and their inflorescences often include 1–3 salverform and never condensed flowers, with ovate to lanceolate calyx lobes.

## Supplementary Material

XML Treatment for Spiradiclis longanensis
